# Breaking barriers: we need a multidisciplinary approach to tackle cancer drug resistance

**DOI:** 10.1038/s44276-025-00129-2

**Published:** 2025-02-27

**Authors:** James Ingham, Jia-Ling Ruan, Matthew A. Coelho

**Affiliations:** 1 Department of Physics, University of Liverpool, Liverpool, UK; 2 Department of Oncology, University of Oxford, Oxford, UK; 3 Cancer, Ageing and Somatic Mutation Programme, Wellcome Sanger Institute, Hinxton, UK

## Abstract

Most cancer-related deaths result from drug-resistant disease(1,2). However, cancer drug resistance is not a primary focus in drug development. Effectively mitigating and treating drug-resistant cancer will require advancements in multiple fields, including early detection, drug discovery, and our fundamental understanding of cancer biology. Therefore, successfully tackling drug resistance requires an increasingly multidisciplinary approach. A recent workshop on cancer drug resistance, jointly organised by Cancer Research UK, the Rosetrees Trust, and the UKRI-funded Physics of Life Network, brought together experts in cell biology, physical sciences, computational biology, drug discovery, and clinicians to focus on these key challenges and devise interdisciplinary approaches to address them. In this perspective, we review the outcomes of the workshop and highlight unanswered research questions. We outline the emerging hallmarks of drug resistance and discuss lessons from the COVID-19 pandemic and antimicrobial resistance that could help accelerate information sharing and timely adoption of research discoveries into the clinic. We envisage that initiatives that drive greater interdisciplinarity will yield rich dividends in developing new ways to better detect, monitor, and treat drug resistance, thereby improving treatment outcomes for cancer patients.

## Introduction

Cancer drug resistance is estimated to account for ~90% of cancer-related deaths [1, 2]. Despite significant advancements in the development of cancer treatments, drug resistance remains a critical challenge, ultimately resulting in ineffective treatments and poor patient outcomes [3]. The heterogeneous nature of cancer [4] calls for an equally diverse range of expertise and interdisciplinary collaborations to address the challenge of drug resistance. Cancer Research UK (CRUK), the Rosetrees Trust, and the UKRI Physics of Life Network organised a workshop on “Tackling Drug Resistance in Cancer” in January 2024, which built upon the success of a similarly interdisciplinary workshop on metastasis in 2021 [5]. The goal was to foster interdisciplinary collaborations and highlight future research challenges to a broader range of disciplines that might usually be associated with a meeting such as this, with the intention of stimulating some novel thinking that would ultimately improve treatment outcomes. The disciplines represented in the audience included clinicians, mathematicians, physicists, chemists, bioinformaticians, engineers, and a range of biological scientists. The workshop was split into four themes: mechanisms of resistance, monitoring of resistance, developing therapies that tackle resistance, and translating new approaches into the clinic. These broad topics span fundamental research into the mechanisms of cancer drug resistance, the development of diagnostic and therapeutic tools for drug-resistant cancers and overcoming challenges in translating basic research into treatments.

This perspective reviews the key themes of the workshop, including the emerging hallmarks of drug resistance, and highlights potential avenues for collaborative efforts to efficiently address these challenges. We will discuss insights gained from multidisciplinary approaches and the importance of considering commercialisation and regulatory processes in academic research. Additionally, we consider lessons learned from the COVID-19 pandemic, where strategies like community-wide data sharing and rapid clinical translation of research discoveries proved pivotal. The global fight against antimicrobial resistance (AMR) provides parallels and insights into addressing cancer drug resistance. AMR underscores the necessity of cross-cutting initiatives, including public health organisations, pharmaceutical companies, clinicians, policymakers, and researchers. Addressing cancer drug resistance similarly requires collective efforts to bridge gaps between basic science, clinical practice, and healthcare infrastructure.

## Lessons from the COVID-19 pandemic

Cancer drug resistance is a manifestation of Darwinian evolution at the cellular level [6]. This process has many parallels with how pathogens evade destruction by the immune system and antimicrobials. Dr. Bartek Waclaw, a physicist at the University of Edinburgh, has worked extensively on the modelling of both antimicrobial resistance and drug resistance in cancer including developing mathematical approaches to understanding the role of growth and migration in the genetic heterogeneity of tumours. He highlighted that there is an opportunity to take successful approaches to the management and treatment of pathogens and apply them to the challenge of drug resistance in cancer. The COVID-19 pandemic called for a coming together of genomic surveillance, the National Health Service, the UK government, and regulatory bodies with open data sharing to rapidly deliver new vaccines and treatments. For example, The COVID-19 Genomics UK (COG-UK) Consortium was an initiative jointly funded by the UK government, UK Research and Innovation (UKRI), and Wellcome to sequence viral genomes from around 600,000 infected individuals by June 2021, thereby mapping variant evolution and outbreaks across the UK. Sharing data from viral genome sequencing empowered vaccine development and enabled modelling of the global spread of new variants [7]. Moreover, genomic data was complemented by functional datasets from high-throughput mutational scanning of the SARS-CoV-2 spike protein. In this way, the functional consequence of observed variants on antibody binding or infectivity was determined [8]. This is a useful example of how functional genomics in parallel with clinical genomic surveillance can effectively discover and interpret genetic variants in real-time, thereby guiding patient treatment and drug discovery.

Initiatives to monitor the evolution of cancer genomes under the selection of anti-cancer drugs have been equally important for directing treatments for patients with drug-resistant cancers. For example, the Hartwig Medical Foundation [9] (https://www.hartwigmedicalfoundation.nl/en/) is a large-scale cancer genomic surveillance effort including post-treatment biopsies that facilitates the provision of cancer genomics data to international researchers [10]. Funded by Cancer Research UK, TRACERx [11] is studying 815 individual lung cancers and will continue to provide valuable insights into cancer genome evolution and the role of tumour heterogeneity in cancer progression [12, 13]. Mathematical models of these evolutionary tumour trajectories may enable more extensive use of computer simulations of different treatment strategies. Over the next decade, TRACERx EVO will map out the effect of cancer variants in early and late-stage cancers and therapy response in around 450 patients with lung cancer. To complement these studies, CRISPR-based approaches to install somatic and germline cancer variants, such as base editing [14–16] and saturation genome editing [17–19] have facilitated the functional characterisation of clinically-observed DNA variants in cancer genomes at unprecedented scale [20]. Recent guidelines on how to incorporate functional assay scores into clinical evidence frameworks for variant interpretation will accelerate the adoption of these data for clinical variant interpretation [21]. The formation of the Atlas of Variant Effects Alliance [22] has catalysed a collaborative effort to generate and share large variant function datasets generated using multiplexed assays of variant effects (MAVEs) [23]. Nevertheless, more work must be done to map the phenotype of DNA variants in the presence of anti-cancer therapies. In this context, benchmarking functional evidence on cancer variants is complicated by the low number of clinical truth-sets i.e. known resistance or variants with no effect on drug sensitivity. In the future, prospective variant-to-function maps in the presence of drug selection pressure [8] will be valuable in generating computational models to predict drug resistance mutations before they emerge in patients [20].

The COVID-19 pandemic saw accelerated assessments by the European Medicines Agency leading to the unprecedented discovery and approval of multiple COVID-19 vaccines in less than a year. In cancer drug discovery, there is an increasing number of accelerated assessments and Priority Medicines designations by the European Medicines Agency (EMA), generally using progression-free survival as a faster interim measure of efficacy before overall survival can be determined [24]. Combination therapies have proven successful in treating HIV and delaying the onset of drug resistance [25]. Similarly, a shift towards drug combinations in cancer could lead to deeper regressions [26]. Moreover, by targeting the same protein with two different molecules, the lower probability of acquiring two distinct drug resistance mutations in the same cell could mitigate or delay the onset of resistance [3]. However, the exploration of targeted drug combinations will require a step-change in how drug companies work together. Promisingly, new guidance from the UK Competitions and Market Authority published in 2023 will make it easier for pharmaceutical companies to collaborate without running the risk of infringing competition law (https://www.gov.uk/government/publications/combination-therapies-prioritisation-statement).

## Lessons from antimicrobial resistance

### Integrating different sectors to increase global awareness

Drug resistance is not limited to cancer. Following the surge of antibiotic development in the early 20th century, antimicrobial resistance (AMR) followed as a major global health challenge in the 21st century. AMR is estimated to cause 5 million infections annually, with particularly high mortality rates in low- and middle-income countries [27]. Cross-country alliances to combat AMR can be traced back to 2009, when the Transatlantic Taskforce on AMR was established, bringing together experts from Canada, Norway, the EU, the UK, and the US to address this threat [28]. In 2015, the World Health Assembly launched the “Global Action Plan on AMR” with five strategic objectives:

1. To improve awareness and understanding of antimicrobial resistance;
2. To strengthen knowledge through surveillance and research;
3. To reduce the incidence of infection;
4. To optimise the use of antimicrobial agents; and
5. To develop the economic case for sustainable investment that considers the needs of all countries, while increasing investment in new medicines, diagnostic tools, vaccines, and other interventions.

This Global Action Plan has since guided the development of national action plans in 178 countries. In the same year, the WHO launched the first global surveillance initiative—the Global Antimicrobial Resistance and Use Surveillance System (GLASS), which provides a standardised framework for sharing country-level AMR data (https://www.who.int/initiatives/glass). Currently, 137 countries participate in GLASS; however, significant data gaps remain, particularly in low-income countries where the burden of AMR is highest.

It took humanity nearly a century to recognise the urgency of the antimicrobial resistance (AMR) crisis. Cancer therapy, which emerged only a few decades after antibiotics, might follow a similar trajectory and face comparable socio-economic challenges. To address the growing issue of cancer drug resistance, a comprehensive action plan is needed. This plan should focus on raising awareness, enhancing surveillance and data sharing across countries, reducing resistance rates, optimising the use of existing therapies, and investing in innovative interventions. Tailoring these efforts to the unique socio-economic needs of individual countries will be essential for overcoming these challenges effectively.

The AMR crisis highlights how environmental factors—such as overuse of antibiotics in agriculture and human medicine—create selective pressures for resistant pathogens. Similarly, cancer drug resistance is exacerbated by external factors like suboptimal dosing, necessitating careful stewardship of therapeutic regimens. Policies encouraging more effective use of cancer therapies, akin to antimicrobial stewardship programmes, could mitigate resistance development. AMR strategies, like rotating antibiotics or using combination therapies, provide useful analogies for cancer treatment. Both fields benefit from adaptive approaches that anticipate resistance patterns and optimise therapeutic impact. Challenges include the need for robust predictive models and the ability to implement adaptive strategies in clinical practice. Investment in data-driven technologies and collaboration between researchers and clinicians will be critical in addressing these issues and achieving practical implementation of adaptive therapies.

## The hallmarks of cancer drug resistance

**Fig. 1:**
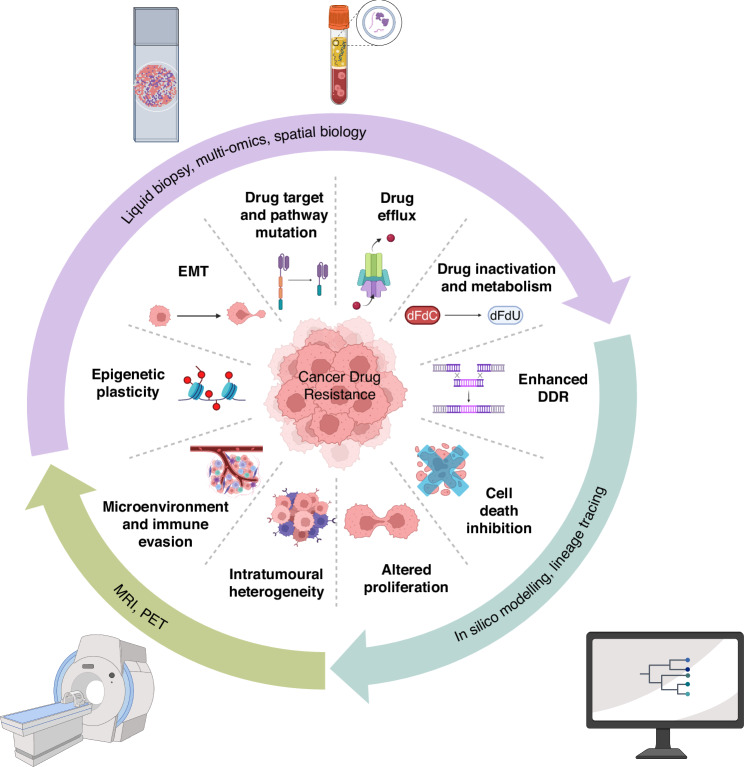
The emerging hallmarks of cancer drug resistance as highlighted by the workshop. This diagram illustrates the key functional characteristics that tumours acquire to become resistant to treatment. The inner circle represents the hallmarks of drug resistance, such as genomic instability, altered drug metabolism (drug efflux and drug inactivation), enhanced DNA damage repair (DDR), mutation of drug targets or signalling proteins, and phenotypic plasticity, including epithelial to mesenchymal transition (EMT). The outer circle highlights some of the diverse approaches used to study these characteristics, including advanced imaging techniques, liquid biopsy and genomics, histology and spatial biology, AI-driven data analysis, and computational modelling. These methodologies are crucial for understanding the mechanisms of drug resistance and developing more effective therapeutic strategies.

The rapid evolution of cancer therapies has been met with an equally rapid adaptation of tumour cells. A deeper understanding of cancer biology has led to the identification of key hallmarks of cancer drug resistance, as explored during the workshop (Fig. 1). These hallmarks, building upon the original hallmarks of cancer [4, 29–31], and the initially proposed hallmarks for anti-cancer drug resistance [32], represent the functional characteristics that tumours acquire to evade anti-cancer treatments. Addressing these hallmarks requires an in-depth understanding of the underlying mechanisms, innovative approaches to therapy, and proactive strategies to mitigate resistance. Here, we summarise some of the key hallmarks and discuss the relevant challenges to address them.

### Intrinsic and acquired resistance

Cancer cells within a tumour exhibit significant heterogeneity, allowing them to inherently possess or acquire capabilities to resist drugs. Intrinsic resistance stems from pre-existing characteristics of the tumour cells, while acquired resistance evolves through genetic or epigenetic changes during therapy. The heterogeneity of tumours makes it difficult to find a one-size-fits-all solution. This resistance can arise from genomic instability and mutations, which alter drug targets or genes in the same signalling pathway, affect the expression of drug transporters [33], or influence drug metabolism and DNA damage repair (DDR) pathways. Currently, research is ongoing to identify specific genetic mutations and pathways involved in drug resistance. Studies, like those by Dr. Louise Fets (MRC London Institute of Medical Sciences), highlight the importance of transporter expression in drug resistance [34].

Efforts to address these challenges have advanced significantly, with precision medicine at the forefront. Genomic sequencing enables early identification of resistance mechanisms, facilitating the tailoring of treatment strategies to individual tumour profiles. Continuous adaptation of cancer cells requires constant updates to treatment strategies. Therefore, in addition to developing therapies to kill cancer effectively, it would be pivotal to invest in research focusing on the underlying mechanism of resistance and developing corresponding preclinical and mathematical models. For example, by utilising diverse genomic information, Prof. Ross Cagan (Wolfson Wohl Cancer Research Centre, University of Glasgow) has developed personalised “fly avatars” to model clinically drug-resistant conditions and identify therapies tailored to individual patient’s tumours [35]. In addition, a mouse model of lung cancer has recently uncovered the role of APOBEC mutagenesis in accelerating the acquisition of drug resistance to EGFR inhibitors [36].

### Epigenetic modifications and environmental factors

Unlike genomic changes, epigenetic modifications, including DNA methylation and histone modification, are reversible and can influence the expression of resistant gene programmes [37]. Drugs targeting epigenetic modifiers have been developed and approved for cancer treatment. Due to the dynamic and reversible nature of epigenetic changes, it is crucial to monitor ongoing epigenetic changes and make adjustments accordingly during the course of therapy. One challenge is epithelial-to-mesenchymal transition (EMT), which enables tumour cells to acquire phenotypic plasticity and enhanced stemness via both epigenetic and post-translational regulation, driving metastasis and drug resistance. Prof. Victoria Sanz-Moreno’s work on MAPK inhibitor resistance through cytoskeleton remodelling, underscores the need for targeted approaches addressing pathways like ROCK-myosin II signalling [38].

Environmental factors also contribute to resistance. The low pH and hypoxic conditions within the tumour microenvironment (TME) drive metabolic adaptations that lead to drug inactivation [39, 40]. The uptake of drugs by non-cancerous cells within the tumour can also contribute to resistance [41]. Identification of key biomarkers within TME to guide therapy will be crucial in enhancing drug efficacy. However, the complexity of the TME makes it challenging to target effectively and generate representative preclinical models. Recent developments in spatial transcriptomics, multiplexed imaging, and imaging mass cytometry, provide a comprehensive view of the molecular landscape and interactions within the TME. Dr. Xiao Fu’s (Cancer Research UK Scotland Institute) integration of computational modelling and experimental lineage tracing exemplifies how these techniques can enhance preclinical models and improve treatment design.

Despite these advances, challenges remain in replicating the complexity of TME in preclinical models and translating findings to clinical settings. Continued investment in spatial and computational techniques will be essential to overcome these barriers.

### Real-time monitoring

Effective management of drug resistance requires early detection and continuous monitoring of tumour evolution. Several imaging and examination techniques used to track cancer treatment responses clinically can directly monitor the hallmarks of drug resistance. Dr. Harpreet Hyare’s interdisciplinary imaging group at University College London uses multiparametric and multidimensional positron emission tomography/magnetic resonance imaging (PET/MRI) to highlight metabolic alterations and intratumoural heterogeneity [42–44]. Advances in machine learning have further enhanced imaging processing, allowing for more efficient detection of substructures that indicate cancer drug resistance. Additionally, liquid biopsies offer a minimally invasive and personalised way to track cancer drug responses over time. Dr. Florent Mouliere’s research demonstrates how circulating tumour DNA can reveal genomic, epigenomic, and microenvironmental signatures, providing a comprehensive view of the tumour’s status over time.

The challenge lies in achieving high sensitivity and specificity in these monitoring techniques, particularly for early detection of resistance. Multimodal integration of data from liquid biopsies and imaging is a promising approach to overcome these limitations, enabling more personalised and adaptive treatments.

### Preclinical modelling and computational approaches

A significant point raised during the workshop was the observation that certain mechanisms present in patients are not replicated in models, prompting the question: how can one determine the validity of an experimental model? An effective model should accurately reflect the human condition, reliably predict clinical outcomes, and be validated against clinical data. Additionally, it should offer mechanistic insights and demonstrate responses to interventions that are consistent with clinical observations. Integrating various preclinical models in cancer drug resistance studies is essential, as each model type has unique strengths that address the limitations of others. *In silico* models provide precise and rapid predictions but may not capture the full complexity of living systems. *In vitro* cell cultures offer detailed insights into cellular mechanisms but lack whole-organism interactions. Animal studies offer a comprehensive system-level understanding but may not fully translate to human biology.

Key challenges include ensuring the validity of preclinical models and alignment with clinical data. Collaboration between computational biologists, clinicians, and experimental researchers is crucial to refine these models and maximise their utility.

### Adaptive therapies

Given the spatial and temporal heterogeneity of cancer drug resistance, adaptive therapies that evolve with the real-time condition of tumours are essential. Digital pathology and multi-omics approaches enable the integration of spatial and genomic data, offering a deeper understanding of tumour dormancy and resistance mechanisms. Dr. Maria Secrier’s (University College London) deep learning model for characterizing cancer dormancy illustrates the potential of these technologies [45]. Adaptive therapy approaches, such as those developed by Dr. Alexander Anderson, a senior mathematician who holds the chair of integrated mathematical oncology at the Moffitt Cancer Center in the USA, prioritises model-guided treatment scheduling over traditional maximum tolerated dose (MTD) strategies. These methods delay resistance and improve efficacy by considering the dynamic nature of tumour evolution [46].

Challenges include the need for robust predictive models and the ability to implement adaptive strategies in clinical practice. Investment in data-driven technologies and collaboration between researchers and clinicians will be critical to addressing these issues and achieve practical implementation of adaptive therapies.

## Accelerating the translation of research into clinical practice

The efficient translation of multidisciplinary research into clinical application is crucial for advancing medical science and improving patient outcomes [5, 47]. The multidisciplinary nature of drug development was a recurring theme throughout the workshop, with presentations highlighting the use of mathematical and experimental modelling [34, 48–50], the application of machine learning and artificial intelligence [51–53], and the translation of research into novel medical devices [54]. Addressing the significant challenges in drug discovery and development requires diverse expertise, including biology, physics, chemistry, mathematics, and data science, to produce actionable outcomes, underscoring the essential role of multidisciplinary collaboration in academia. However, multidisciplinary research can pose challenges, such as difficulties in integrating diverse methodologies, communicating effectively across disciplines, and aligning varying research priorities. Workshops such as the ‘Tackling drug resistance in cancer,’ where an interdisciplinary cohort of experts was brought together, represent a key step needed in addressing these difficulties by fostering collaboration and understanding across fields.

### Improving cross-sector communication

A significant challenge in effective multidisciplinary research is integrating data and techniques from diverse sources, including genomics, proteomics, functional assays, advanced imaging techniques, and machine learning models, into actionable clinical insights. The relative lack of standardised cross-institutional frameworks for data sharing and interpretability, especially across disciplines, often impairs collaboration, leads to duplication of resources, and slows progress. The advancement of new approaches, such as federated learning, enables a secure and decentralised method for data integration while maintaining patient privacy and research independence [55, 56]. This may overcome some of these challenges but will require broader community support and adoption.

Prof. Trevor Graham (Institute of Cancer Research) presented the application of mathematical modelling to genomic and molecular pathology [57]. This emphasised the importance of considering drug resistance throughout the drug development process, especially at the early stages. Using the KRAS G12C inhibitor sotorasib as an example [58], Prof. Graham illustrated how rapidly drug resistance can undermine the clinical efficacy of promising treatments if not thoroughly considered within the development process. This work highlights the transformative potential of integrating mathematical modelling into drug development, offering a blueprint for anticipating and mitigating resistance mechanisms early, a strategy that could be widely adopted across the field to enhance the durability of emerging therapies.

### Academia and industry – working towards shared goals

A prominent discussion point centred on the increasing need for collaborations between academia and industry to establish effective pathways for translating research into clinical applications. Despite shared goals, significant gaps often arise from differing priorities and timelines between academic research and industry developments. Prof. Tim Eisen (University of Cambridge and AstraZeneca) provided insight into how academic and industry research groups could collaborate more effectively. Academics often focus on securing funding to tackle ambitious new challenges, which can result in lengthy research timelines. In contrast, the industry often has shorter research timelines and milestones, which can be aligned with shorter funding cycles for start-ups. Establishing clear joint objectives from the outset would benefit both groups and help facilitate more effective collaborations and translation of research with clinical impact. This discussion highlighted the critical need for effective collaborations between academia and industry to bridge the gap between foundational research and practical applications and accelerate the development of impactful therapies that address key clinical challenges. One successful example of this is Open Targets (https://www.opentargets.org), an international consortium of pharma and academic partners facilitating rapid exchange of expertise and capabilities. Initiatives such as Open Targets should be expanded, and similar programs should be developed where applicable and needed. The workshop highlighted the importance of strengthening bidirectional cross-sector integration. This included supporting PhD projects and university teaching that incorporates industry-focused aspects, as well as additional industry-funded fellowship positions to tackle shared challenges across academia and industry.

### Regulatory hurdles

The successful translation of research also requires engaging stakeholders beyond academia and industry; otherwise, the relevance and scalability of any outcomes may be limited. For example, engaging clinicians during the design phase of diagnostic tools or treatment methodologies can ensure compatibility with clinical workflows, and patient input can help identify usability challenges that might otherwise be overlooked. Early stakeholder engagement aligns research objectives with broader societal needs, improving the chances of successful translation. Initiatives incorporating public involvement and patient engagement, such as focus groups or advisory boards, are increasingly becoming key to research proposals as they have proven important in improving outcome relevance and usability [59].

Once a successful product or procedure for treating a specific disease has been developed, its transition into clinical practice must also overcome significant regulatory barriers. These barriers vary across regional regulatory approval bodies, often involving specific requirements that impose substantial time and financial hurdles on researchers and companies looking to translate their research into clinical applications. Lessons learned from the COVID-19 vaccine approvals demonstrate how adaptive regulatory frameworks can be both rigorous and efficient, suggesting a template for future cancer therapies. Within the workshop, Prof. Peter Weightman discussed his experience in commercialising the Liverpool Diagnostic Infrared Wand, a novel medical device incorporating infra-red spectroscopy and AI. He highlighted the key advantages of tackling both the technical and regulatory aspects in parallel, as well as the early involvement of regulatory experts, particularly since regulation is a prolonged process that requires experience not always common among academics. This emphasised the importance of early collaboration with regulatory experts in aligning research designs and protocols to meet the future approval criteria needed for approval, facilitating smoother transitions to clinical trials and, ultimately, commercialisation. This discussion underscored the inherently multidisciplinary nature of such medical advancements, demonstrating how integrating physics, medical science, data science, and regulatory expertise was essential to begin translating innovative research into practical clinical applications.

## Conclusions and future research directions

Addressing the key challenges to tackle cancer drug resistance will require a multifaceted approach and enhanced collaboration across diverse fields. This will need experts from non-biological or medical disciplines—including the physical sciences, mathematics, and data sciences – and the application of advanced analytical techniques, mathematical modelling, and data-driven insights to elucidate the complexities of drug resistance. This interdisciplinary approach to investigating cancer drug resistance is a shared goal for Cancer Research UK and the UKRI Physics of Life Network. To accelerate this, we propose three future research initiatives.

### Encouraging cross-sector collaboration and data sharing

Enhancing collaborations between academia and industry would accelerate the identification of drug resistance mechanisms by allowing the investigation of molecules early in the drug discovery process and maximise the use of diverse skills and resources. The success of these integrated research efforts will rely on the open and early dissemination of functional and clinical data on drug resistance across the scientific community. Finally, collaborative drug discovery projects that leverage the strengths of academia, industry, and regulatory bodies could drive the rapid development of effective therapies.

### Drug combinations and second-line therapies

Combination therapies, which target multiple hallmarks of cancer drug resistance, may enhance cancer cell eradication and reduce the likelihood of developing drug resistance. This approach has proven successful in treating pathogens like bacteria and HIV. For instance, combining immunotherapy with targeted therapies [60], or using combinations of different targeted therapies could represent effective future treatment approaches. While some progress has already been made in this area, significant advancements are still needed. Current efforts include integrating combination therapies into clinical practice and optimising their design. Key areas for further development include refining preclinical models to better predict drug combination efficacy, improving clinical trial design to evaluate these combinations effectively, and ensuring that clinical studies are designed to address the complexity of resistance mechanisms.

### Implementing artificial intelligence (AI) to predict drug resistance

AI models enable the analysis of vast multimodal and multidimensional datasets, identifying patterns and mutations associated with resistance [61]. By integrating AI into the analysis of clinical data (liquid biopsy, imaging, histopathology analysis), oncologists could make complex decisions based on multiparametric data, thereby helping to detect cancers earlier and tailoring treatments to minimise the development of therapy resistance. A future aim would be to predict drug resistance mechanisms to drugs before they emerge in the clinic.

In summary, tackling cancer drug resistance requires a proactive, collaborative approach that integrates new technologies, encourages data sharing, and fosters partnerships across academia, industry, and healthcare practitioners. Anticipating drug resistance before its clinical manifestation will be crucial to reduce the likelihood of treatment failures. By taking on these three initiatives as a research community, we can develop more effective strategies to combat drug resistance and improve the efficacy of cancer treatments.

## Data Availability

No datasets were generated or analysed during the current study.
